# Decoding *Bacillus* spp.: Antimicrobial Diversity, Biocontrol Mechanisms, and Safe Deployment in Plant Disease Management

**DOI:** 10.3390/plants15121834

**Published:** 2026-06-13

**Authors:** Sajad Ali

**Affiliations:** Department of Biological Sciences, College of Science, King Faisal University, Al-Ahsa 31982, Saudi Arabia; sabhat@kfu.edu.sa

**Keywords:** *Bacillus*, antimicrobial compounds, biocontrol agents, phytopathogens, biopesticides

## Abstract

Chemical agents have long been used to control plant diseases, but their effects on the environment and lack of alignment with sustainable development goals are making them gradually unsuitable. One trend in green agriculture is the use of *Bacillus* species for the biocontrol of plant diseases. Due to their vast metabolic and genetic diversity, *Bacillus* spp. can contribute significantly to the soil ecosystem, while also enhancing plant resilience to biotic and abiotic stresses. *Bacillus* spp. are widely used in the agrobiotech industry due to their multi-functional versatility and are well-known for protecting plants from numerous plant diseases. In this review, we discussed the diversity and functions of antimicrobial compounds (AMCs) produced by *Bacillus* spp., along with their roles in plant growth promotion (PGP), and immunity. Furthermore, we highlighted the potential of *Bacillus* spp. as biopesticides in host plants, ways to enhance their biocontrol efficacy, and also addressed their possibility to cause disease in host plants. Considering the beneficial impacts of *Bacillus* spp. on PGP and pathogen biocontrol and their disease-causing capability, we discussed the possible solutions for a safe development of *Bacillus*-based biocontrol agent (BCA). Collectively, these insights can guide the selection of *Bacillus* strains with broad-spectrum or target-specific activity against pathogens, ensuring minimal adverse effects on the host.

## 1. Introduction

Plant diseases pose a major risk to worldwide food security, reducing crop yield and quality, thereby causing substantial economic losses. It is estimated that approximately 15% of unprotected crops worldwide are lost annually due to plant diseases caused by numerous plant pathogens [1]. These pathogens, including viruses, bacteria, fungi, nematodes, and parasites, cause a wide range of plant diseases that lead to substantial crop losses worldwide [2,3]. Although the use of agrochemicals, such as herbicides, insecticides, fungicides, and rodenticides, has historically contributed to higher crop productivity and quality, these chemical compounds are deliberately introduced into the environment to target living organisms and may pose environmental and human health risks [4]. Growing concerns over pesticide residues and their impacts have driven a shift toward more sustainable and environmentally conscious crop management practices in recent years [3].

Biological management through the use of beneficial microbes offers a promising approach to control plant diseases, strengthening plant immunity and promoting environmental sustainability [3]. Microbial BCAs, often isolated from the phyllosphere, endosphere, or rhizosphere bacteria, play a key role in suppressing plant pathogenic microorganisms by preventing infection and limiting pathogen establishment in host plants [3,5]. Recently, bacteria have gained increased attention due to their intimate association with host tissues and enhanced resilience to both biotic and abiotic stresses. Root-associated endophytes are known to synthesize phytohormones, such as auxins and gibberellins, which contribute significantly to growth and development [6,7]. Among the microbes, *Bacillus* spp. have emerged as promising BCAs because of their ability to suppress plant pathogens through multiple mechanisms, including competition for nutrient and space, modification of plant surface characteristics, production of AMCs, disruption of disease progression, and activation of systemic resistance (SR) in plants [5,7,8]. However, some *Bacillus* spp. have also been reported to exert harmful effect on plants, animals and humans depending on species, host, and different environmental conditions [9,10]. Therefore, it is also essential to understand the factors contributing to *Bacillus*-induced pathogenicity across different hosts before employing these bacteria as biofertilizer or BCA in agricultural systems. This review highlights the role of *Bacillus* spp. in plant growth and immunity, the bioactive metabolites they synthesize, their potential as BCAs, their pathogenic attributes, and future research prospective.

## 2. Role of *Bacillus* spp. in Plants

Numerous bacteria have characteristics that promote plant development, thereby enhancing agricultural productivity and increasing resistance to various diseases and environmental stressors [11]. *Bacillus* is one of the most studied plant growth-promoting rhizobacteria (PGPR). In a study on spore-forming bacteria for the biocontrol of *Heterodera glycines* on soybean, approximately 92.6% of PGPR that caused mortality of *H*. *glycines* belong to the genus *Bacillus* [12]. Based on diverse biochemical traits, such as the production of lipopeptides and polyketides (PKs), potassium solubilization, proteolytic and amylolytic activity, siderophore production, and biofilm formation, numerous *Bacillus* spp. have been identified as promising plant growth promoters [6].

*Bacillus* spp. are capable of synthesizing siderophores, typically forming halo zones ranging from 2.5 to 5.33 mm. Recent findings demonstrated that *B. licheniformis* BaDB06 in *Lessertia frutescens* contributed for 24% siderophores formation, whereas *B. subtilis* ZIM3 was the greatest producer with 26% contribution [11]. Additionally, *Bacillus* spp., particularly *B. subtilis*, facilitate nodulation by encouraging the colonization of symbiotic bacteria [13]. *Bacillus* spp. also contribute to plant nutrition through the solubilization of inorganic nutrients and the production of volatile organic compounds (VOCs) [5]. VOCs, such as acetoin (3-hydroxy-2-butanone) and 2,3-butanediol produced by *B*. *subtilis*, modulate cytokinin and ethylene (ET) homeostasis, resulting in enhanced plant growth. Applying 2,3-butanediol, a VOC produced by *B. subtilis*, significantly increases leaf size in *Arabidopsis thaliana* [14]. *B*. *cereus* (ADJ1) isolated from *Agave desmettiana* Jacob showed strong PGP properties, including high production of indole-3-acetic acid (IAA) (9.46 µgml^−1^), ammonia (64.67 µmol mL^−1^), zinc solubilization, ACC deaminase production, and biofilm formation. Furthermore, it also enhanced nitrogen fixation, hydrogen cyanide generation, and enhanced wheat germination and seedling development under salt stress [15]. Similarly, seeds of wheat cv. Pakistan-13 treated with *B. methylotrophicus* demonstrated increased biomass, longer roots, and improved vegetative shoot growth under salt stress [16]. Although variability exists among strains, these findings collectively suggest that siderophore production in *Bacillus* spp. typically falls within a moderate functional range, indicating that PGP is likely driven by a combination of traits rather than a single dominant mechanism.

Plant growth and agricultural productivity are seriously threatened by global climate change, which is made worse by environmental conditions like drought, salt, greenhouse gas emissions, and extremely high temperatures. *Bacillus* spp. help the plants to withstand abiotic stress [17]. Across cultivars, salinity, and seasons, the use of endophytes, such as *B. firmus* J22N and *Bacillus* sp. REN51N increased peanut pod and haulm yield by 14–19%. In peanuts, the inoculation of endophytes in seeds coated with charcoal-based carrier (10^9^–10^10^ CFU/g carrier) resulted in increased potassium absorption, decreased levels of phenol and H_2_O_2_, and increased proline accumulation in saline water (1.5–2.0 dS/m) [17]. Some *Bacillus* strains exhibit strong growth-promoting effects, especially under stress. For instance, *B. cereus* PK6-15, *B. subtilis* PK5-26, and *B. circulans* PK3-109 significantly enhanced *A. thaliana* growth under salt stress [18]. When compared to uninoculated plants under salt stress,* B. circulans* PK3-15 and PK3-109 boosted plant fresh weight by more than 50%; however, they did not stimulate plant growth under normal circumstances [18]. These findings suggest that some *Bacillus* spp. could be promising BCAs only under stress conditions and thereby the specific stress-tolerant trait of *Bacillus* spp. could be useful in specific stressed areas. Moreover, different *Bacillus* spp. were also reported to be associated with stress response and antioxidant gene upregulation [19]. According to another study, *B. megaterium* CACC109 and CACC119 strains demonstrated a number of activities that promote plant growth, such as nitrogen fixation, phosphate solubilization, siderophore secretion, IAA production, 1-aminocyclopropane-1-carboxylate deaminase activity, and exopolysaccharide production. Following inoculation, CACC109 and CACC119 enhanced root development in both stressed and non-stressed circumstances and markedly enhanced rice (*Oryza sativa* L.) seed germination under osmotic stress (LB liquid medium supplemented with polyethylene glycol 6000 at concentrations of 0, 10, and 20%, respectively, and mannitol with 0, 150, and 200 mM, respectively) [19]. Their application upregulated genes associated with antioxidant activity (e.g., *OsCAT*, *OsPOD*, *OsAPX*, and *OsSOD*) and genes that respond to drought (e.g., *OsWRKY47*, *OsZIP23*, *OsDREB2*, *OsNAC066*, *OsAREB1*, and *OsAREB2*) increased in the treated plants [19].

Various *Bacillus* spp. produce plant growth regulators during their interactions with plants, including gibberellins, ET, abscisic acid, cytokines, and IAA, which have a substantial influence on the processes of plant growth and development [20]. In *A. thaliana*, VOCs synthesized by *B. subtilis* OKB105 can control auxin homeostasis, causing elevated auxin levels in the roots and lesser auxin levels in the leaves. This redistribution may be able to provide the best possible plant growth since auxin restricts leaf expansion while encouraging root development [21]. It is known that *B. subtilis* is capable of producing phytohormones on its own. For example, inoculating lettuce plants with *B. subtilis* (capable of producing cytokinin) greatly boosted the concentration of cytokinin in plants and improved plant growth and development. Over the course of eight days, the cytokinin-producing strain of *B*. *subtilis* enhanced lettuce growth, increasing shoot and root biomass by approximately 30% [22]. In both treated and untreated tomato plants, *B. velezensis*, *B. megaterium*, and *Herpaspirillum huttiense*, either separately or in combination, reduced the levels of hydrogen peroxide (H_2_O_2_) and malondialdehyde (MDA) and enhanced the activities of antioxidant enzymes [23]. The production of enzymes, such as superoxide dismutase (SOD), glutathione reductase (GR), catalase (CAT), ascorbate peroxidase (APX), lipid peroxidase (POD), and H_2_O_2_ content in leaves, as well as the uptake of potassium in peanuts, were significantly modulated in another study [17]. Salinity raised the activities of the enzymes that scavenge reactive oxygen species (ROS) and endophytes like *B. firmus* J22N, *B. tequilensis* SEN15N, and *Bacillus* spp. REN51N further boosted their activities [17]. The role of *Bacillus* spp. having plant growth-promoting and immunity-boosting capability has been summarized in Table 1.

## 3. *Bacillus* spp. as BCAs in Plants

**Figure 1:**
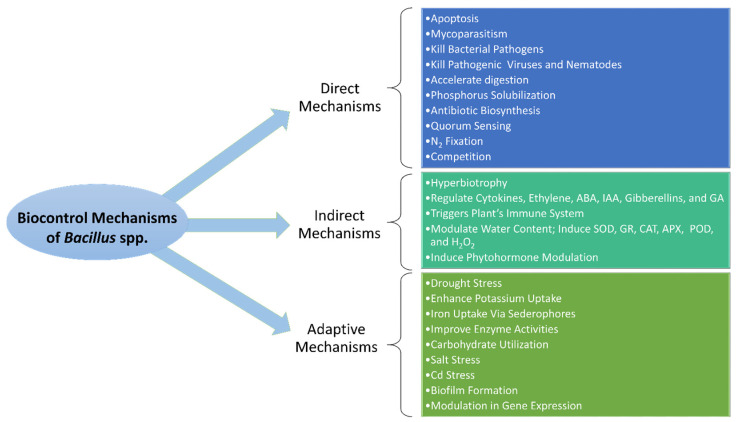
Biocontrol mechanisms of *Bacillus* spp. in plant growth promotion (PGP) and pathogen control. The figure illustrates their direct, indirect and adaptive effects against pathogens, modulation of plant immune responses and phytohormones, and enhancement of plant tolerance to abiotic stresses.

Numerous studies have examined the role of *Bacillus* spp. in PGP and crop disease management. Several *Bacillus* spp. have been identified as effective BCAs for controlling a wide range of diseases in major crops [5]. The application of *Bacillus* spp. enhances plant growth and suppresses bacterial, fungal, nematode, and viral diseases without posing environmental risks. In the rhizosphere, plant–microbe interactions enable *Bacillus* spp. to form biofilms and produce secondary metabolites, such as fengycin, iturin, bacillomycin, and surfactin, which collectively suppress plant pathogens [3,5]. As shown in Figure 1, *Bacillus*-mediated biocontrol involves a combination of direct antagonism, competition, and induction of plant immune responses, emphasizing the multifaceted nature of pathogen suppression.

**Figure 2:**
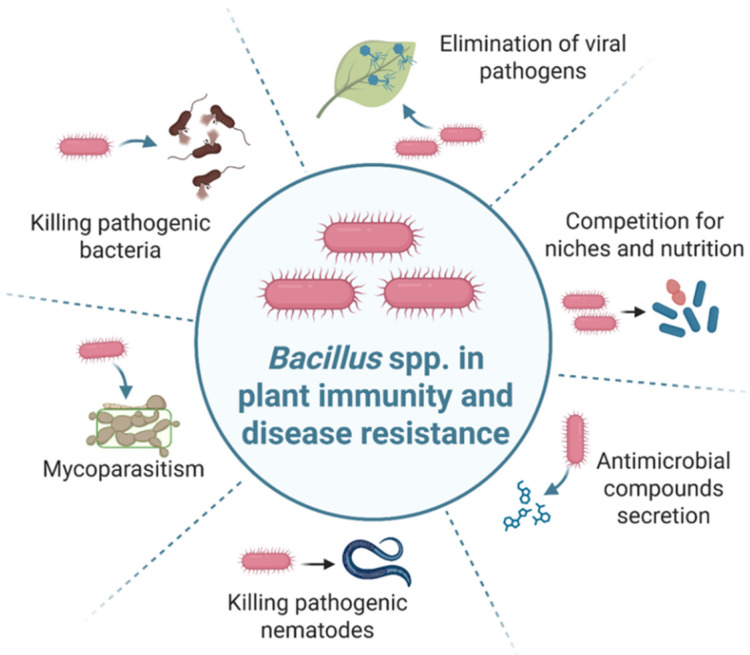
Overview of *Bacillus* spp. mediated plant protection through multiple antagonistic and immunomodulatory mechanisms. *Bacillus* species enhance plant immunity and disease resistance through diverse strategies including production of antimicrobial compounds, competition for nutrients and niches, and suppression of pathogenic microbes and exhibit mycoparasitism, nematode inhibition, and antiviral activities, contributing to overall plant health and resilience.

Additionally, *Bacillus* spp. are widely utilized in agricultural research as promising BCAs [5]. Figure 2 summarizes the diverse agricultural applications of *Bacillus* spp., ranging from disease suppression to microbiome modulation and PGP.

### 3.1. Biocontrol of Bacterial Pathogens

*Bacillus* spp. have demonstrated strong biocontrol efficacy against bacterial plant pathogens through direct antagonism and host-mediated resistance. For instance, *B*. *velezensis* D significantly suppressed *Ralstonia solanacearum*, the causal agent of tobacco bacterial wilt, while simultaneously enhancing host resistance under greenhouse conditions [30]. Similarly, *B. velezensis* SS-38.4 effectively colonized the phyllosphere of sugar beet and reduced disease severity caused by *Pseudomonas syringae*, highlighting the importance of ecological fitness in successful biocontrol [31]. In another study, *B*. *velezensis* MJ23 producing antimicrobial lipopeptides exhibited strong antibacterial activity against *Xanthomonas oryzae* pv. *oryzae*, disrupting pathogen cell membranes and inhibiting biofilm formation [32]. Recent genomic and functional studies further indicate that *Bacillus* strains deploy diverse mechanisms, including antibiotic production, competition for nutrients, and induction of SR, enabling broad-spectrum suppression of bacterial pathogens [33]. Collectively, these findings emphasize that effective biocontrol by *Bacillus* spp. is not solely dependent on AMCs but also on their ability to colonize plant niches and modulate host immunity. *Bacillus* spp. also modulate the microbial composition in the rhizosphere, thereby suppressing the pathogens. For instance, inoculation with *B*. *atrophaeus* DX-9 increased the relative abundance of *Nitrobacter*, *Agrobacterium*, and *Bradyrhizobium* [34]. *B*. *velezensis* JZ also suppressed *B*. *altitudinis* m-1, the causal agent of strawberry leaf spot, mainly by increasing intracellular ROS and decreasing Ca^2+^-ATPase activity, without significantly altering cell membrane permeability [35]. In cherry plants, *Bacillus* strains WY66 and WY519 induced genes associated with jasmonic acid (JA), ET, and salicylic acid (SA) pathways, thereby suppressing *Agrobacterium tumefaciens*, the pathogen responsible for crown gall [36]. Taken together, these findings indicate that *Bacillus* strains employ distinct but converging mechanisms including oxidative stress induction, ion transport disruption, and phytohormone signaling to suppress pathogens, highlighting a coordinated multi-target mode of action rather than a single dominant pathway. Although *Bacillus* inoculation can significantly influence rhizosphere and endosphere microbial assemblages, current evidence indicates that many of these effects are transient and highly context-dependent. Long-term studies across multiple cropping cycles and crop rotations are still needed to determine whether inoculation-induced microbiome shifts persist, are reversible, or result in stable alterations of indigenous microbial community structure and function.

### 3.2. Biocontrol of Fungal Pathogens

*Bacillus* spp. act as BCAs controlling different fungal pathogens in plants [2]. Some *Bacillus* strains also enrich beneficial microbial communities while reducing pathogen populations, thereby enhancing plant growth [37]. For example, *B. velezensis* NT35 significantly reduced the relative abundance of *Ilyonectria*, *Fusarium*, *Neonectria*, and *Dactylonectria* pathogens causing root rot and rusty root rot in ginseng and promoted beneficial rhizosphere bacteria, including *Luteimonas*, *Nocardioides*, *Sphingomonas*, and *Gemmatimonas* [37]. This strain enriched members of Sphingomonadales, Sphingomonadaceae, and Actinomycetes and inhibited the mycelial growth of *I*. *robusta* by 94.12% at 10^7^ CFU/mL. Sporulation and spore germination inhibition reached 100% and 90.31% at 10^4^ and 10^8^ CFU/mL, respectively [37]. The high biocontrol efficiency observed for *B. velezensis* NT35 suggests that effective biocontrol is strongly associated with simultaneous modulation of both pathogen suppression and rhizosphere microbial restructuring. This indicates that community-level shifts, rather than direct antagonism alone, play a critical role in disease suppression. *Bacillus* spp. were also found effective in reducing postharvest diseases and regulating their gene expression [38,39]. Gray mold caused by *Botrytis cinerea* is a major disease of tomato, and *Bacillus* spp. have shown significant potential in suppressing soil-borne fungal pathogens. *Bacillus* strains exhibited antagonistic activity against *Rhizoctonia solani*, *Sclerotium rolfsii*, and *Fusarium oxysporum* f. sp. *ciceri*, which cause root rot, collar rot, and wilt in chickpea (*C. arietinum* L.) respectively [40]. Members of the *B*. *amyloliquefaciens* complex (*B*. *amyloliquefaciens*, *B*. *velezensis*, *B*. *nakamurai*, and *B*. *siamensis*) are recognized as promising BCAs for fungal disease control [41]. For instance, *B*. *amyloliquefaciens* YN201732 produces lipopeptides, particularly bacillomycin D and fengycin, that effectively suppress *Erysiphe cichoracearum*, the agent of tobacco powdery mildew [42].* B. amyloliquefaciens* SQ-2 inhibited more than 40% of mycelial growth in all tested grape varieties, with 53% inhibition in Kyoho grapes. SEM analysis confirmed severe morphological damage to *Aspergillus tubingensis* [43]. Additionally, *B*. *amyloliquefaciens* SFB-1 suppressed *C*. *fimbriata* by inducing mycelial swelling, completely inhibiting spore germination at 10^8^ CFU/mL, and reducing mycelial growth by 81.01% [38]. Transcriptome profiling revealed 1164 DEGs associated with cell wall integrity, membrane stability, germination, detoxification, and oxidative stress [38]. For postharvest anthracnose in mango caused by *Colletotrichum gloeosporioides*, *B*. *siamensis* significantly reduced disease severity and enhanced fruit quality, with transcriptomic evidence indicating upregulation of genes involved in plant–pathogen interactions, hormone signaling, and resistance compound biosynthesis [39].

### 3.3. Biocontrol of Nematodes

Several *Bacillus* spp. have also shown strong nematicidal activity (Table 2). *B*. *velezensis* VB7 caused 96.66% juvenile mortality and 87.95% egg hatching inhibition in *Meloidogyne incognita* after 96 h. The strain activated MAMP-triggered immunity by upregulating *WRKY*, *LOX*, *PAL*, *MYB*, and *PR* genes [44]. *B. velezensis* YS-AT-DS1 (Bv-DS1) exhibits nematicidal activity against *Meloidogyne incognita* with a 71.62% mortality rate in second-stage juvenile (J2s). The combined effects of Bv-DS1 on protecting plants against RKN and promoting their growth may be linked to the control of water and solute transport through TIP (tonoplast intrinsic protein). Therefore, in sustainable agriculture, the Bv-DS1 strain may be employed as a BCA for RKN control [45]. Strains *B*. *aryabhattai* Ba1-7, *B*. *megatherium* Ba2-4, and *B*. *halotolerans* Ba2-6 effectively controlled *Heterodera glycines* and enhanced soybean growth, with Ba2-6 inducing SR through upregulation of R1, PR3a, PR5, and NPR1-2 linked to SA and JA pathways [46]. VOCs from *B*. *cereus* G5 showed strong fumigant activity against *M*. *graminicola*, reducing gall formation and nematode populations. The VOCs stimulated defense-related genes in SA (*OsNPR1*, *OsWRKY45*, and *OsPAL1*), JA (*OsJaMYB*, *OsAOS2*), and ET (*OsACS1*) pathways [47]. Compounds, such as allomatrine, morantel, 1-octen-3-ol, and 3-methyl-2-butanol, exhibited potent nematicidal activity, with 1-octen-3-ol showing an LC_50_ of 758.95 mg/L against *M*. *graminicola* J2s [47].

### 3.4. Biocontrol of Viral Diseases

Although *Bacillus* spp. are primarily recognized for their antibacterial and antifungal activities, accumulating evidence indicates that they can also suppress plant viral diseases through multiple direct and indirect mechanisms. One of the best-characterized mechanisms involves the induction of systemic resistance (ISR), whereby *Bacillus*-derived metabolites activate salicylic acid (SA), jasmonic acid (JA), and ethylene (ET) signaling pathways, leading to enhanced expression of pathogenesis-related (PR) genes and reduced viral accumulation [30,84]. Treatment with *B. subtilis* strains has been reported to significantly reduce disease severity caused by Tobacco mosaic virus (TMV), Cucumber mosaic virus (CMV), and Tomato-spotted wilt virus (TSWV) through activation of host defense responses [82,83,90]. Lipopeptides such as surfactin, fengycin, and iturin have also been implicated in antiviral defense through membrane-associated signaling events that enhance plant immune responses and stimulate the accumulation of defense-related metabolites [84,91]. Furthermore, *B*. *amyloliquefaciens* and *B. velezensis* strains have been shown to increase antioxidant enzyme activities, including superoxide dismutase, catalase, and peroxidase, thereby mitigating virus-induced oxidative stress and limiting symptom development [91,92]. Recent transcriptomic studies further suggest that *Bacillus*-mediated antiviral activity involves extensive reprogramming of hormone-regulated defense networks and RNA-silencing-associated pathways, resulting in reduced viral titers and enhanced plant resilience [82]. In addition to ISR, several *Bacillus* species produce extracellular ribonucleases (RNases) capable of degrading viral RNA molecules, thereby interfering with viral replication and systemic movement. In greenhouse trials, *B*. *subtilis* 26D and Ttl2 enhanced tomato resistance to potato virus X (PVX) and potato virus Y (PVY) by releasing RNases and phytohormones, reducing viral accumulation, restoring fruit yield, and induced systemic resistance (ISR) via activation of SA- and JA-related genes. They also modulated hormone levels, reducing abscisic acid accumulation in PVY infection and increasing abscisic acid in PVX-infected plants, thereby improving resistance [93]. Collectively, these findings demonstrate that *Bacillus* spp. suppress viral diseases through a combination of RNase-mediated viral RNA degradation, lipopeptide-triggered immune priming, antioxidant regulation, and ISR activation, highlighting their potential as sustainable biological tools for managing economically important plant viruses.

However, as research continues, several *Bacillus*-based BCAs are commercially available for controlling soil-borne pathogens and protecting crops (Table 3), while many others are under evaluation for future deployment.

## 4. Secondary Metabolites Synthesized by *Bacillus* spp. for Agricultural Applications

*Bacillus* spp. synthesize structurally varied secondary metabolites with broad antimicrobial activity [5]. Based on biosynthetic origin, *Bacillus* peptide antibiotics are categorized into nonribosomal peptide synthetases (NRPSs) and ribosomally synthesized peptides (RSPs) [5]. Moreover, *Bacillus* spp. also produce some antibacterial and antifungal VOCs [96].

### 4.1. Nonribosomal Peptides

NRPSs, such as gramicidin, tyrocidine, bacitracin, surfactin, iturins, and fengycins, are produced through multienzyme thiotemplate systems that select and condense amino acid residues. Following synthesis, these peptides may undergo further modifications, such as heterocyclic ring formation, acylation, glycosylation, or N-methylation [5,7,97]. *Bacillus* lipopeptides represent a major group of NRPSs, notably the surfactin, iturin, and fengycin families. Surfactin is a heptapeptide containing D-configured residues at positions 3 and 6, with applications in biocontrol, food preservation, medicine, and bioremediation, although commercial use remains limited by low yields and high production costs [98,99]. This class of antimicrobial peptides (AMPs), which are produced by various *Bacillus* spp., includes linchenysin, pumilacidin, and WH1fungin [98,99]. Moreover, *B. subtilis* species produce a family of antifungal lipopeptide substances called iturinic, which includes iturin, bacillomycin D, bacillomycin F, bacillomycin L, mycosubtilin, and mojavensin [100,101]. The fengycin, another family of antifungal lipopeptides, comprising fengycin, plipastatin, and Agrastatin1, is characterized by a specific peptide sequence linked to a C14–C18 β-hydroxy fatty acid [102]. Fengycin exerts its antifungal effects by damaging cell walls and membranes, disrupting metabolic activities, inducing programmed cell death and autophagy, and activating plant defense responses [95]. Although these lipopeptides share amphiphilic properties, their membrane targets and mechanisms of action differ substantially. Surfactins primarily interact with phospholipid bilayers, causing membrane destabilization and increased permeability through detergent-like effects, but generally exhibit weaker direct antifungal activity. In contrast, iturins display a strong affinity for ergosterol-containing fungal membranes, where they induce pore formation, ion leakage, and cell death. Fengycins preferentially interact with fungal membrane phospholipids and lipid microdomains, resulting in membrane deformation and disruption of filamentous fungal growth, while exerting comparatively limited effects on most bacterial membranes. These differences in membrane specificity contribute to the complementary antimicrobial spectra of *Bacillus* lipopeptides [95,97].

Recent work continues to uncover new non-ribosomally synthesized lipopeptides (e.g., Baelezcin A), a novel cyclic lipopeptide (C_52_H_91_N_7_O_13_) produced by *B*. *velezensis* SJ100083. Structural elucidation using ultrahigh-performance liquid chromatography quadrupole Orbitrap high-resolution mass spectrometry (UHPLC-Q-Orbitrap-HRMS) and nuclear magnetic resonance (NMR) confirmed its composition. Application of Baelezcin A at 25 mg/L significantly reduced both the incidence and severity of cherry gray mold caused by *Botrytis cinerea* [103]. It caused the accumulation of ROS within the spores and the trickle of mycelium cytoplasmal contents, which caused decreased spore germination [103]. Regardless of the lipopeptides, there are other NRPSs as well. It has been demonstrated that some strains of *B*. *subtilis* and *B*. *licheniformis* release the non-ribosomal peptide bacitracin, which inhibits the cell wall of Gram-positive bacteria [104]. The N-terminal alanine residue and L-anticapsin in the small peptide bacilysin, which is released by *B. subtilis*, *B. amyloliquefaciens*, and *B. pumilus*, have antibacterial action against different plant pathogenic bacteria [5]. Under iron-limited circumstances, a number of *Bacillus* spp. produce bacillibactin and petrobactin [105]. *Paenibacillus larvae* synthesizes the tripeptide antibiotic, sevadicin, consisting of D-phenylalanine, D-alanine, and tryptophan via an NRPS-encoded gene cluster, which exhibits antibacterial activity [106]. Phosphonate natural compounds synthesized non-ribosomally by *Bacillus* have been another promising source of salable pesticides. Recently two new phosphonates were reported, produced by *B. velezensis* for agricultural use. Isolation and structure clarification discovered a novel di- and tripeptide made of l-alanine and a C-terminal l-phosphonoalanine, which were named phosphonoalamides E and F [107].

### 4.2. Polyketides (PKs) and Hybrid Metabolites

**Figure 3:**
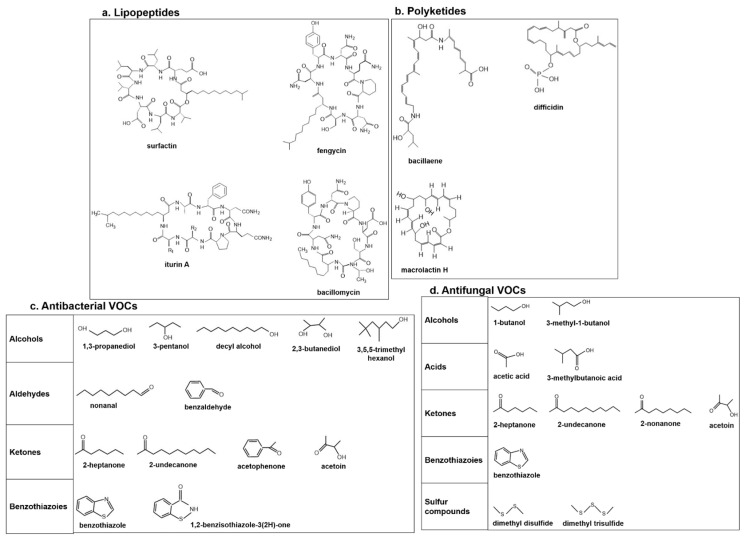
Key AMCs produced by *Bacillus* spp. (**a**) Main lipopeptides, (**b**) PKs produced by *Bacillus* spp., (**c**) VOCs showing antibacterial activity against phytopathogenic bacteria, (**d**) VOCs showing antifungal activity against phytopathogenic fungi.

*Bacillus* spp. also produce PKs, a class of RSP-associated antimicrobial and anticancer compounds. Major PK compounds produced by *Bacillus* spp. are bacillaene, difficidin, and macrolactin [108]. They have a wide variety of structures and are usually cationic (Figure 3).

The underlying metabolic pathways including the gene clusters encoding polyketide synthases (PKSs) are now being increasingly well-understood [7]. For example, Bacillaene is encoded by *pksX* gene cluster [109]. The antifungal PK basiliskamide was produced by *B. laterosporus* [110]. The synthesis of PKs by *B. amyloliquefaciens* FZB42 has been thoroughly investigated, and three PKS gene clusters (*pks1*, *pks2*, and *pks3*) have been identified. These clusters play a role in the biosynthesis of difficidin/oxydifficidin (*pks3*), macrolactins (*pks2*), and bacillaene (*pks1*, also called *pksX*) [111]. Recent research has reported new PKs produced by *Bacillus* spp. Chemical investigation of the organic extract of *B. velezensis* MBTDLP1 is characterized as a novel antimicrobial agent that mimics macrocyclic PK showing prospective antibacterial potential against methicillin-resistant *S. aureus* (MIC 0.38 µg/mL) and is presented by positive control chloramphenicol (6.25 µg/mL) [112]. Additional examples of RSP antibiotics made by *Bacillus* spp. include subtilins, ericins, mersacidin, and sublancin 168 [113]. A recent study showed *B. safensis* APC 4099 to be a potential BCA. Genomic study of *B. safensis* APC 4099 discovered biosynthetic gene clusters encoding numerous AMPs and secondary metabolites. In particular, RSP safencin E, a new, anionic, circular bacteriocin that is 6 kDa in size, was shown to be 52.5% identical to butyrivibriocin AR10. Moreover, gene clusters encoding RSPs including pumilarin and plantazolicin, pumilacidin A, bacilysin, and bacillibactin were also identified in *B. safensis* APC 4099 [114]. As BCAs for food ecosystems against spoiling and pathogenic bacteria, *B. safensis* and its bioactive substances provide a remedy to the worldwide issue of the 1.3 billion tons of agricultural produce wasted each year [114].

### 4.3. Volatile Organic Compounds (VOCs)

In addition to nonribosomal peptides and polyketides, *Bacillus* spp. produce a diverse range of VOCs that contribute to pathogen suppression, plant growth promotion, and induction of host defense responses. Unlike diffusible metabolites, VOCs can act over long distances through the gaseous phase, enabling interactions with pathogens and plants without direct physical contact [14]. *Bacillus*-derived VOCs mainly include alcohols, ketones, aldehydes, sulfur-containing compounds, pyrazines, and aromatic compounds, many of which originate from amino acid catabolism, pyruvate metabolism, and fatty acid degradation pathways. Among the best-characterized VOCs are acetoin (3-hydroxy-2-butanone) and 2,3-butanediol, produced through pyruvate fermentation pathways. These compounds enhance plant growth by modulating phytohormone signaling and inducing systemic resistance [14,21]. VOCs produced by *B. subtilis* have been shown to increase biomass accumulation in *A. thaliana* and alter auxin homeostasis, thereby promoting root development and stress adaptation [14,21]. In addition to growth-promoting effects, several ketones, aldehydes, and sulfur-containing volatiles exhibit direct antimicrobial activity by disrupting membrane integrity, inhibiting spore germination, and interfering with pathogen metabolism. Recent studies have also demonstrated that *Bacillus* VOCs possess nematicidal activity. For example, VOCs produced by *B. cereus* G5, including 1-octen-3-ol and 3-methyl-2-butanol, significantly reduced *Meloidogyne graminicola* populations and activated SA-, JA-, and ET-associated defense pathways in rice [47]. A recent study reported a novel formaldehyde dehydrogenase in *B. subtilis* that detoxifies formaldehyde, which is toxic for all living forms, creating a favorable environment for other beneficiary microbes in the rhizosphere and within plants [96]. Collectively, VOCs represent a mechanistically distinct class of antimicrobial metabolites that complement lipopeptides and polyketides in *Bacillus*-mediated biocontrol (Table 4). Identifying such novel compounds and knowing their beneficial effects may open a new era for agricultural evolution. Figure 3 illustrates the structural diversity of major antimicrobial metabolites produced by *Bacillus* spp., highlighting the distinction between lipopeptides, PKs, and VOCs which differ in target specificity.

## 5. Improving the Biocontrol Efficacy of *Bacillus* spp.

Practical applicability and expansion of BCAs are typically constrained by their inconsistent prevention performance in field settings. The colonization and functional efficiency of inoculated *Bacillus* spp. can be influenced by a variety of complex and dynamic variables, including soil properties, plant genotypes, and native microbiome [115]. As a result, several strategies have been tried to increase their effectiveness. These include applying bacterial isolates and chemical agents (such as bactericides) together, using antagonistic metabolites in place of or together with BCAs, and combining *Bacillus* strains with organic ingredients to create bioorganic fertilizers [115,116,117]. Two new approaches are synthetic microbial consortia (SynCom) and rhizosphere-derived prebiotics, which have been compiled to increase the biocontrol effectiveness of *Bacillus* spp. on various levels [118].

The term “SynCom” describes a community made up of a small number of strains with various roles. A well-designed SynCom can demonstrate more metabolic variation, environmental adaption capacity, and broader/stronger activities as compared to individual BCAs [67]. Compared to a single strain, coinoculation of the BCAs *B*. *velezensis* BN8.2, *Pseudomonas chlororaphis* subsp. *piscium* PS5, and *Trichoderma virens* T2C1.4 considerably reduced banana *Fusarium* wilt [119]. A recent study shows that the particular combination of *B. velezensis* AP197 and *B. velezensis* AP298 can improve the biological control efficiency of several plant diseases [120]. At present, rational SynCom building typically adheres to the design–build–test–learn (DBTL) cycle and may be accomplished using either a “top down” or “bottom up” methodology. The process of gradually selecting important microbes from environmental samples by physical and functional analysis is known as the “top down strategy” [121]. A recent study revealed the main rhizobacteria species that reduce maize seed-borne *Fusarium* were identified by investigation using progressive dilution and rhizodepositional attraction. In order to create a simplified SynCom, a bacterial consortium comprising eight strains from the genera *Bacillus*, *Burkholderia*, *Enterobacter*, *Lysobacter*, *Stenotrophomonas*, *Pseudoxanthomonas*, *Pseudomonas*, and *Acinetobacter* was built using these crucial strains and modified based on higher environmental stability and disease suppression capacity. SynCom is more efficient than a single strain and randomly generated microbiota at inhibiting seed-borne *Fusarium* [122]. An alliance of five bacterial isolates for the control of fungal pathogen in *Nicotiana attenuata*, including *P. azotoformans* A70, *P. Frederiksbergensis* A176, and *Arthrobacter nitroguajacolicus* E46 with *B. megaterium* B55 and *B. mojavensis* K1 may develop improved biofilms over time. Strain K1 synthesizes the antifungal compound surfactin, which inhibits fungal growth [123]. These highlighted the conceptual basis of SynCom for creating synthetic microbiome with exceptional ecological stability and biocontrol efficacy. A “bottom up” strategy for increasing *Bacillus* spp. biocontrol efficacy aims to optimize circumstances at the microbiological level. This entails enhancing the innate biocontrol systems of the bacteria as well as their interactions with the pathogen and plant. Important tactics include boosting the synthesis of advantageous metabolites such as enzymes and antibiotics, improved nutrient uptake, and encouraging advantageous plant–microbe interactions [121]. In order to create a “bottom up”-based SynCom against two *Fusarium* pathogens, a previous study chose a consortium of four strains that include *B. amyloliquefaciens*, *Serratia marcescens*, *P. fluorescens*, and *Rahnella aquatilis* from 150 bacterial isolates. Consequently, both *Fusarium* pathogens were considerably suppressed by the microbial consortium, which was consistently more effective than the individual isolates [124].

**Figure 4:**
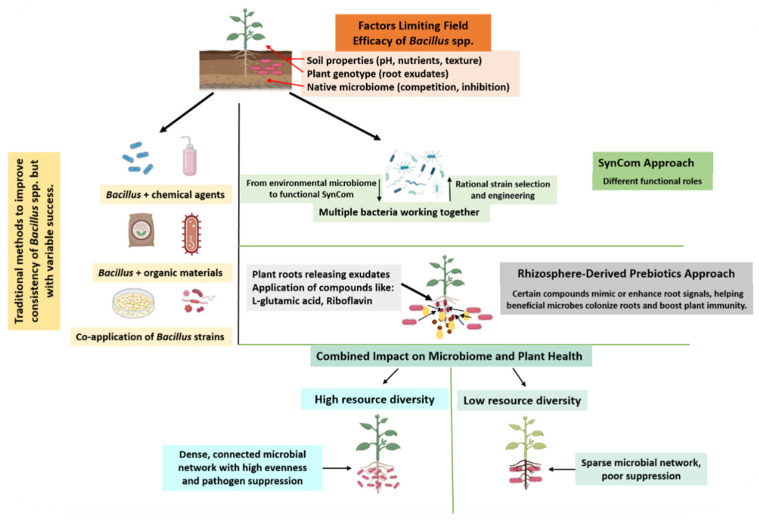
Strategies enhancing field performance of *Bacillus*-based BCAs. The left side of the figure shows the traditional methods to improve the biocontrol efficacy of *Bacillus* spp. like using chemicals with *Bacillus* spp. or using different *Bacillus* spp. The right side of the figure shows modern SynCom and rizosphere-derived prebiotics approaches and combines impact of microbiome to boost the biocontrol efficacy of *Bacillus* spp.

When used directly in the field, BCAs, which are identifiable as unique invading species, typically cannot adapt to the local soil matrix or interact with the local microbiota, which results in poor biocontrol efficiency and ineffective rhizosphere colonization [125]. Certain chemicals can be created as prebiotics to improve root colonization and biocontrol efficiency because specific signals emitted from root exudates or root deposits attract *Bacillus* spp. and trigger their activities [118]. The external supplementation with L-glutamic acid, which changed the rhizosphere microbiota and enhanced the major taxa in the strawberry anthosphere, and also enhanced the proportions of *Bacillaceae*, *Streptomyces*, and *Burkholderiaceae* in the tomato rhizosphere, significantly lowered diseases caused by *Botrytis* and *Fusarium* in both habitats [125]. Riboflavin was recognized as a multifunctional prebiotic that enhances the biocontrol efficacy of *B. subtilis* Tpb55 against tobacco black shank induced by *P. nicotianae* by stimulating the activity of CAT, POD, SOD, and β-1,3-glucanase in the roots of Tpb55-inoculated tobacco seedlings [126]. The augmentation of resource variety modified trophic network structure, enhanced microbial evenness, and thus improved the reliability of effective pathogen management. On the other hand, the consequences of poor resource variety on invading resistance were more diverse and less effective. Therefore, plant disease suppression was greatly aided by increases in the evenness and connectivity of dominant species brought about by high resource diversity. Additionally, microbial processes relevant to the control of the plant immune system were elevated by high carbohydrate diversity [127]. Figure 4 depicts the role of rhizosphere-derived signals and prebiotics in enhancing *Bacillus* colonization and activity, providing a framework for improving field-level biocontrol consistency.

To face real-world challenges, it is important to select or engineer strains that maintain functional activity under abiotic stress. Recent studies highlight that stress-tolerant *Bacillus* (e.g., *B. subtilis*, *B. amyloliquefaciens*) capable of producing stable lipopeptides (such as surfactin) and forming robust biofilms exhibits enhanced persistence under drought, salinity, and temperature fluctuations [128,129]. Climate-resilient *B*. *velezensis* strains (e.g., TL7, S1) demonstrate stable, broad-spectrum antifungal activity, enhanced plant growth, and environmental stress resilience, making them highly suitable for sustainable, climate-smart agriculture by maintaining performance across variable field conditions [130]. At the same time, modern formulation strategies have significantly improved the survival, shelf life, and field persistence of *Bacillus*-based BCAs. Microencapsulation using polymers such as alginate, chitosan, carrageenan, and gum-based matrices protects spores from UV radiation, desiccation, and oxidative stress while enabling controlled release. For instance, alginate or natural gum microcapsules containing *B. velezensis* achieved up to 90% pathogen inhibition and improved long-term shelf life the stability in soil [131]. Similarly, spray-dried encapsulated formulations increased spore survival upto 95% in *B. thuringiensis* subsp. *kurstaki* IMBL-B9 compared to non-encapsulated cells and maintained increased survival rate and storage stability at 54 ± 2 °C for up to 6 weeks [132,133]. Slow-release macrosphere systems using chitosan–carrageenan further enhanced disease control in crops such as Chinese cabbage by improving bacterial persistence and delivery efficiency. It further improved persistence by ensuring gradual microbial release and sustained metabolite production, making them particularly suitable for long-duration field applications [134]. Consequently, seed coatings are most appropriate for early-season protection, whereas microencapsulated and slow-release systems are preferred for improving storage stability and maintaining consistent field performance under variable environmental conditions [135]. These advances demonstrate that formulation optimization is central to translating lab efficacy into field reliability. Moreover, integrating *Bacillus*-based BCAs with crop rotation, reduced chemical inputs, and organic amendments has been shown to improve consistency and long-term efficacy. Large-scale agricultural programs, particularly in Brazil, demonstrate that combining multifunctional *Bacillus* strains with agronomic management practices enhances both disease suppression and crop productivity under field conditions [136].

## 6. *Bacillus* spp. as Opportunistic Pathogens

**Figure 5:**
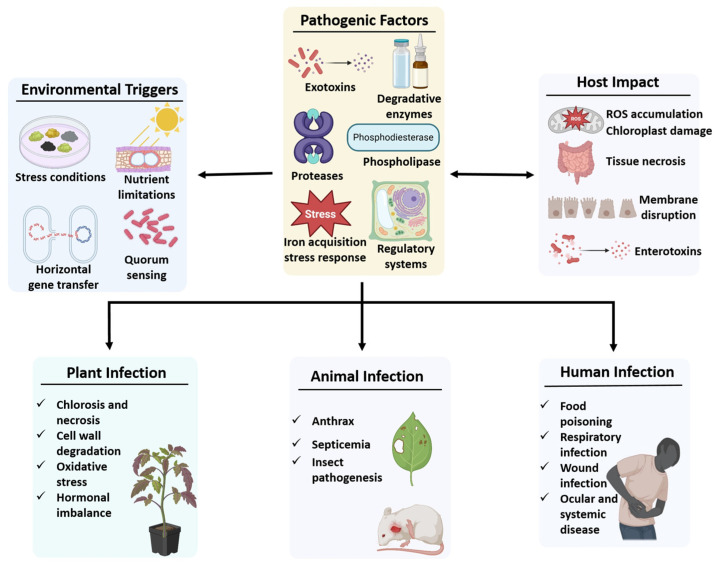
Cross-kingdom opportunistic pathogenicity of *Bacillus* spp.: environmental triggers, virulence determinants, and host physiological disruption. The schematic illustrates how environmental stressors (nutrient limitation, abiotic stress, and horizontal gene transfer) can activate pathogenic determinants in Bacillus spp., including toxin complexes (Hbl, Nhe, CytK), degradative enzymes (proteases, phospholipases, cell wall-degrading enzymes), and regulatory systems controlling iron acquisition and oxidative stress responses. These factors mediate physiological and molecular disruptions across plant, animal, and human hosts, leading to oxidative stress, membrane damage, tissue necrosis, and systemic disease manifestations.

Although many *Bacillus* species are recognized for their role as plant growth-promoting rhizobacteria (PGPR) and BCAs, a growing body of evidence highlights their potential as opportunistic pathogens in plants, animals, and humans [137,138,139]. This dual lifestyle reflects a remarkable ecological and evolutionary plasticity within the *Bacillus* genus, driven by genetic diversity, horizontal gene transfer, environmental triggers, and host–microbe interactions (Figure 5) [140]. Effective evaluation of *Bacillus* in agricultural systems, therefore, requires an integrated understanding of both beneficial traits and pathogenic potential.

### 6.1. Physiological Disruption in Plants

In plant systems, opportunistic pathogenicity may manifest through *Bacillus*-induced physiological disruptions, especially under favorable conditions such as high inoculum load, stressed hosts, or weakened immune responses [140]. Fruit rot on muskmelon (*Cucumis melo*) was reported to be caused by *B. pumilus* in China. When bacterial rot in onion bulbs was found in South Korean warehouses in 2008, *B. amyloliquefaciens* was found to be the causative agent of onion bulb [141]. Reisolated from inoculation onion bulbs, the isolated bacteria produced the identical rot symptom as was observed in naturally infected onions after storage [141]. *B. altitudinis* has recently been found to be a cause pomegranate seed rot in China [142]. Pathogenic determinants such as cell wall-degrading enzymes, proteases, and phospholipases can compromise plant cellular integrity by degrading structural components, disrupting membranes, and inducing oxidative imbalance [143]. Such disruptions can lead to chloroplast ultrastructure damage, impaired photosynthesis, lipid peroxidation, and accumulation of ROS, ultimately manifesting as necrotic lesions or chlorosis in infected tissues [144]. The interplay between ROS and host antioxidant defenses shapes disease progression. Production of ROS beyond the buffering capacity of enzymes like SOD, CAT, and POD can intensify tissue damage [145]. In contrast, beneficial *Bacillus* strains often enhance antioxidant capacity and prime defense pathways, highlighting how different strain genotypes yield divergent physiological outcomes [146]. Recent advances indicate that ROS and reactive nitrogen species (RNS) function not only as indicators of oxidative stress but also as central signaling molecules that coordinate plant immune responses. ROS–RNS crosstalk regulates redox homeostasis, antioxidant enzyme activities, defense-related gene expression, hypersensitive responses, and programmed cell death through complex signaling networks spanning chloroplasts, mitochondria, peroxisomes, and the apoplast [147]. Within this framework, *Bacillus*-mediated modulation of ROS accumulation and antioxidant defenses can be viewed as part of a broader redox signaling cascade that integrates hormonal pathways, stress perception, and pathogen defense. Beneficial *Bacillus* strains often promote balanced ROS production and enhanced antioxidant capacity, whereas opportunistic pathogenic strains may trigger excessive oxidative stress and cellular damage. Thus, the contrasting effects of different *Bacillus* species on host redox status likely reflect their differential influence on ROS–RNS signaling networks that ultimately determine plant resistance or susceptibility. Comparative studies on stress signatures between benign and opportunistic strains deepen our mechanistic understanding of how specific virulence determinants outweigh host defenses [145,148]. The predominant bacteria responsible for peach fruit rot was *B. velezensis* zk1. It damaged the chloroplasts, mitochondria, respiratory chain function, and associated free radical scavenging enzyme systems. MDA levels rose as a result of cell death, but levels of vitamin C, dialdehyde, flavonoids, and total phenols fell. Activities of ammonia lyase, polyphenol oxidase, SOD, CAT, POD, and APX also declined [10].

### 6.2. Opportunistic Pathogen in Human and Animals

There have also been reports of several *B. subtilis* strains causing illness in other living forms. For instance, the virulence genes of *B. subtilis* G7 isolated from a deep-sea hydrothermal vent are abundant and capable of killing fish and mice [149]. Before being recognized as a human pathogen that causes both intestinal and extraintestinal disorders, *B. cereus* was thought to be innocuous for almost 80 years [150]. Numerous toxins, such as the pore-forming toxins hemolysin BL (HBL) and nonhemolytic enterotoxin (NHE), have been linked to illness. HBL binds to the mammalian surface receptors LITAF and CDIP1, and both HBL and NHE cause potassium efflux and trigger the NLRP3 inflammasome, which results in pyroptosis [150]. Common intestinal disorders caused by *B. cereus* include diarrhea, vomiting, and nausea. Nevertheless, it has been linked to severe infections in immunocompromised hosts and can result in endophthalmitis, which may lead to blindness, and septicemia [151]. Highly resistant spores produced by *B. anthracis* may spread to grazing animals and linger in the environment for decades. Uncoagulated blood leaking from natural openings and acute or hyperacute septicemia cause anthrax to cause abrupt death without any outward clinical symptoms. Infected animals, carcasses, or animal products are typically the source of infection in humans [152]. The Centers for Disease Control and Prevention (CDCs) state that sudden death is a common symptom in animals, with a recent study reporting 998 deaths out of 6354 instances. Depending on the kind of anthrax, the fatality rate in people varies; inhalational anthrax is the most lethal, with death rates as high as 50% [8]. Numerous diseases, including bacteremia, septicemia, wound infections, and endocarditis, can also be brought on by other *Bacillus* spp., such as *B. cereus*, *B. subtilis*, and *B. licheniformis*, especially in immunocompromised people [153]. Some proteins produced by some *Bacillus* spp. are responsible for molecular mimicry and evade the host immune system. Sphingomyelinases (SMase) are phosphodiesterases found in bacterial and mammalian cells that catalyze the hydrolysis of sphingomyelin (SM) [154,155]. Pathogenic bacteria like *B. cereus* and *B. anthracis* generate SMase C, one of the bacterial sphingomyelinases, which breaks down the ester link between ceramide and phosphocholine. Its catalytic function is identical to that of human neutral sphingomyelinase 2 (nSMase2) [154,156]. Numerous physical and clinical activities, including membrane dynamics, cellular signaling, migration, colonization, immune system evasion in the initial phases of infections, and infection development, are facilitated by SMase C [154,155,156]. *B. thuringiensis*, famous for insecticidal Cry toxins, has also caused opportunistic infections in immunocompromised animals and humans, indicating that host range can extend beyond target pests under some conditions [157]. Strains classified as *B. cereus* biovar *anthracis* have recently emerged as anthrax-like pathogens by acquiring plasmids analogous to those of *B. anthracis* [138]. This confirms that virulence in *Bacillus* can be conferred through plasmid acquisition and recombination, illustrating dynamic genomic plasticity driving pathogenic phenotypes [138,158].

### 6.3. Environmental Triggers of Pathogenic Shift

Pathogenicity in *Bacillus* is not strictly determined by taxonomy but can be triggered by environmental and host factors [140]. Temperature stress, nutrient limitation, and host immune suppression create conditions conducive to expression of virulence traits [159]. For example, alternative sigma factor SigB in *Bacillus cereus* under heat shock and nutrient stress conditions reprogram gene expression to enhance survival and potentially virulence-associated traits [159]. Quorum sensing systems modulate expression of toxins and degradative enzymes in response to cell density [160]. Iron acquisition systems and oxidative stress response regulators (e.g., PerR, SigB) are pivotal in enabling survival and virulence under fluctuating abiotic stresses [161]. Environmental stressors may also alter the balance between beneficial and pathogenic outcomes. For example, under drought or heat stress in plants, physiological barriers are compromised, potentially facilitating opportunistic infection by *Bacillus* strains that might otherwise function as beneficial rhizobacteria [157].

## 7. Strategic Selection and Safe Deployment of *Bacillus* spp. as BCAs in Plants

The expanding use of *Bacillus* spp. as BCAs in crop protection requires a rigorous selection framework that integrates genomic safety, physiological performance, and ecological stability [139,162].

### 7.1. Strain-Level Safety Assessment and Selection

Whole-genome sequencing (WGS) has become the cornerstone of safe strain selection [162,163,164]. Comparative genomics enables identification of toxin gene clusters such as *hbl*, *nhe*, and *cyt*K, enterotoxin operons (*hbl*ACD, *nhe*ABC, and *cyt*K) as well as plasmid-borne virulence determinants associated with lineages related to *B. anthracis* and *B. thuringiensis* [139,165]. Moreover, strain-level variation within closely related taxa necessitates exclusion based on genomic content rather than species identity. Recent experimental work demonstrated that strain-specific characterization of *B. velezensis* isolates reveals significant differences in functional gene clusters, supporting the need for genome-guided selection prior to field use [67]. Beneficial strains typically promote antioxidant enzyme activities (SOD, CAT, and POD) and induce systemic resistance (ISR) via JA and ET signaling pathways [3]. Lipopeptides commonly produced by *B. subtilis* and *B. velezensis* are central to antifungal activity and ISR priming without exerting host toxicity [95,97]. Stress-response profiling under variable temperature, osmotic, and nutrient conditions is essential, as environmental stress may activate latent virulence pathways in some strains [159]. Stable expression of beneficial traits across environmental gradients is therefore a key selection criterion. Species with established safety and agricultural performance records should be prioritized. These include *B. subtilis*, *B. amyloliquefaciens*, and *B. velezensis*. These taxa are renowned for producing antimicrobial metabolites, siderophores, and VOCs that suppress fungal pathogens such as *Fusarium*, *Rhizoctonia*, and *Botrytis* spp. [7,97]. However, some strains within these species were also reported pathogenic to plants [10]. Multilocus sequence typing (MLST) and average nucleotide identity (ANI) analyses ensure accurate taxonomic placement and prevent misclassification within the *Bacillus* spp. [163].

### 7.2. Field Deployment and Ecological Monitoring

Formulation technology significantly influences both efficacy and biosafety. *Bacillus* spp. are commonly formulated as endospores due to their environmental resilience [95]. However, inoculum density must be optimized to avoid ecological imbalance or unintended proliferation. Encapsulation, carrier-based granules, and seed-coating formulations enable controlled release and targeted rhizosphere colonization [131,132,134]. Localized soil or seed treatments are preferable to broad foliar applications when targeting soil-borne pathogens, minimizing exposure to non-target environments and reducing ecological risks [166]. Field-scale experiments demonstrated that defined inoculum concentrations of *B. velezensis* effectively controlled target organisms while maintaining controlled population dynamics in complex substrates such as manure systems [166]. Long-term environmental monitoring is essential following field deployment. Horizontal gene transfer (HGT) within soil microbial communities may alter strain characteristics over time. Periodic reisolation and genomic reassessment can confirm genetic stability and absence of acquired virulence factors [164]. Quantitative molecular tools such as strain-specific qPCR enable precise tracking of *Bacillus* populations in the rhizosphere. For example, a recent study developed a TaqMan-based detection system for *B. velezensis* that allowed accurate quantification of colonization dynamics and demonstrated a direct relationship between population stability and controlled biocontrol performance [167]. Such monitoring frameworks are essential to detect unintended proliferation or ecological imbalance and ensure long-term biosafety.

### 7.3. Regulatory and Biosafety Compliance

Regulatory frameworks governing microbial biopesticides require toxicological, environmental, and non-target organism assessments [140]. Transparent genomic documentation, safety assays, and reproducible efficacy data enhance regulatory approval and public acceptance. Where necessary, genome editing approaches may be used to remove undesirable genes, although compliance with biosafety regulations for genetically modified microorganisms must be ensured [140].

Robust biosafety assurance of *Bacillus*-based BCAs is achieved through compliance with established regulatory frameworks that evaluate microbial products prior to commercialization and field deployment. In the United States, the United States Environmental Protection Agency (EPA) regulates microbial biopesticides under the Federal Insecticide, Fungicide, and Rodenticide Act (FIFRA), classifying them as microbial pesticides distinct from biochemical and conventional chemical pesticides [168]. The EPA requires a comprehensive data package including strain identity, manufacturing process, toxicology (acute oral, dermal, and inhalation), pathogenicity/infectivity, and environmental fate. Importantly, microbial agents such as *Bacillus* spp. must undergo Tier I and Tier II ecological risk assessments, which evaluate effects on non-target organisms (e.g., pollinators, aquatic species) and environmental persistence before registration [169]. Similarly, in the European Union, the European Food Safety Authority (EFSA) conducts scientific risk assessments for microbial active substances under Regulation (EC) No. 1107/2009. EFSA classifies microbial BCAs based on taxonomic identity and biological properties, requiring detailed characterization at the strain level, including genome-based identification, absence of virulence factors, and antimicrobial resistance profiling [170]. Risk assessment focuses on human health (toxicity, infectivity), environmental safety (soil persistence, dispersal), and non-target organism effects, supported by experimental data. For example, EFSA evaluations of *B. velezensis* strains have emphasized the need for exclusion of enterotoxin genes and confirmation of non-pathogenic behavior under realistic exposure scenarios [171].

These regulatory systems adopt a multi-tiered, weight-of-evidence approach, integrating laboratory assays, genomic data, and field studies to determine safety. These frameworks ensure that only strains meeting stringent biosafety and ecological compatibility criteria are approved, thereby mitigating risks associated with opportunistic pathogenicity and unintended environmental impacts. A major limitation of current regulatory frameworks is that safety assessments are largely species-based, whereas accumulating evidence indicates that pathogenicity and beneficiality in *Bacillus* are often strain-specific and influenced by environmental conditions. Consequently, future regulatory frameworks should incorporate genomic risk profiling and condition-dependent phenotypic assessments rather than relying solely on taxonomic classification.

## 8. Conclusions and Future Perspectives

*Bacillus* spp. represent a multifunctional platform for sustainable plant disease management, integrating antimicrobial metabolite production, microbiome modulation, and activation of host immune responses. Isolating and applying specific *Bacillus* strains having well-known biocontrol capabilities should be used for specific application, such as *B. velezensis* Y6 for controlling *R. solani* in rice [67]. However, inconsistencies in field performance and the emerging recognition of opportunistic pathogenicity highlight the necessity for precision-based strain selection and biosafety evaluation. The safe selection and deployment of *Bacillus* spp. as BCAs depend on a multi-tiered strategy integrating genomic exclusion of virulence determinants, physiological validation, ecological monitoring, and regulatory compliance. Recognizing the genetic proximity between beneficial strains and opportunistic pathogens underscores the necessity of strain-level precision. An important challenge in the development of *Bacillus*-based BCAs is distinguishing beneficial strains from opportunistic pathogenic relatives. Comparative genomic analyses have shown that beneficial *Bacillus* strains are generally enriched in biosynthetic gene clusters involved in antimicrobial production, rhizosphere competence, biofilm formation, and plant growth promotion, whereas pathogenic strains frequently harbor virulence-associated genes encoding enterotoxins, hemolysins, or other host-damaging factors. Nevertheless, the presence or absence of specific genes alone may not reliably predict ecological behavior, because microbial phenotypes are also shaped by gene regulation, environmental conditions, and host–microbe interactions. Consequently, genomic screening should be complemented by transcriptomic, metabolomic, and phenotypic characterization to ensure accurate biosafety assessment and strain selection. New emerging techniques like CRISPR-Cas9, ZFNs, and TALENs could be applied to make engineered *Bacillus-*based BCAs for better efficacy with broad spectrum applications, while keeping the adverse effect at the minimum level. However, despite their considerable potential, the practical deployment of genetically engineered microbial biocontrol agents remains constrained by stringent regulatory requirements and public acceptance concerns in many jurisdictions, particularly within the European Union (EU), where genetically modified microorganisms are subject to separate and substantially more rigorous approval procedures than conventional microbial biopesticides. Furthermore, linking genome mining with functional metabolomics will accelerate the discovery of novel AMCs. Regardless of the discovery of numerous novel gene clusters of prospective antimicrobials, their parts have not yet been investigated and they are still uncharacterized.

Real-world experiments other than the lab and controlled greenhouse experiments would greatly help in understanding their capability and real-world efficacy which might show further development steps to mitigate these challenges and ensure safe BCA development. The expression of antimicrobial biosynthetic gene clusters in *Bacillus* spp. is strongly influenced by environmental conditions, including soil moisture, temperature, pH, nutrient status, and plant-derived signals. These factors can modulate the production of bioactive metabolites through stress-responsive regulatory pathways and quorum-sensing mechanisms. Consequently, environmental fluctuations may affect the consistency of *Bacillus*-mediated pathogen suppression under field conditions. Although abiotic stress can alter bacterial physiology, colonization dynamics, and secondary metabolite production, there is currently limited evidence that beneficial *Bacillus* strains undergo a transition toward pathogenic phenotypes. Future multi-omics studies are needed to elucidate how environmental stressors regulate biosynthetic gene cluster expression and influence the ecological stability and biocontrol efficacy of *Bacillus* inoculants. Simultaneous transcriptomic and metabolomic profiling of individual strains under plant-beneficial versus stress-induced conditions could reveal key regulatory switches controlling antimicrobial production, host colonization, stress adaptation, and virulence-associated traits. As some *Bacillus* strains can also be pathogenic to plants and animals, precautions should be taken using these stains as BCAs. Machine learning approach in analyzing the specific stain physiology and genomics combining with other multiomic approaches will enable the proper exploitation of *Bacillus*-based BCAs’ fundamental processes and application potential for ecofriendly and sustainable plant disease management.

Although current knowledge of *Bacillus–host* interactions has been largely derived from bulk transcriptomic, metabolomic, and physiological analyses, these approaches often mask the substantial cellular heterogeneity that exists within plant tissues during microbial colonization. Recent advances in single-cell RNA sequencing (scRNA-seq) and spatial transcriptomics have revealed that individual cell types can exhibit distinct transcriptional programs, signaling responses, and immune states during plant–microbe interactions. These technologies have successfully resolved cell-type-specific responses to bacterial, fungal, and viral colonization and uncovered spatially coordinated defense networks that cannot be detected using conventional bulk analyses. A recent review highlights how integrating single-cell and spatial omics can transform our understanding of plant–microbe interactions by linking microbial colonization patterns with host cellular responses at unprecedented resolution [172]. Applying these approaches to *Bacillus*–plant systems represents a promising future direction for elucidating niche-specific colonization strategies, immune modulation, and the molecular determinants that distinguish beneficial endophytes from opportunistic pathogens.

## Figures and Tables

**Table 1:** Role of different *Bacillus* spp. in plant growth and immunity.

*Bacillus* spp.	Function	References
*B. altitudinis* Y-14	Lowered the fruit deterioration rate and quality decay, repressed MDA buildup, improved SOD and POD action levels. 1-methylcyclopropene + *B. altitudinis* Y-14 treatment effectively condensed the deterioration rate.	[24]
*B. australimaris* BLR41	Shoot length was observed by 30% in the medicinal plant *Barleria lupulina* Lindl in the test by zinc and phosphate solubilization.	[6]
*B. licheniformis* BaDB6, *B. velezensis* SM-95	Stimulated seedling development of *Lessertia frutescens* and produce siderophores and hydrolytic enzymes.	[11]
*B. pacificus* G124	Enhanced plant drought tolerance, leaf area, chlorophyll content, relative water content, and root enlargement in both *A. thaliana* and *Medicago sativa* seedlings under barren conditions. Moreover, G124 improved antioxidant enzyme actions and osmolyte gathering, while reducing MDA and ROS levels.	[25]
*B. safensis* P1.5S	Solubilize phosphate under abiotic stress like different pH, temperature, and salinity.	[26]
*B. siamensis* R27	Stimulated lettuce seedling growth and aided removed Cd^2+^ from the growth medium with 80.1% efficacy and improved antioxidant actions for scavenging ROS brought by Cd^2+^ stress.	[27]
*B. subtilis*	Improved wheat (*Triticum aestivum* L.) seed germination in different Cd concentrations.	[28]
*B. subtilis*	*B. subtilis* decreased the buildup of superoxide, improved the plant defense enzymes in chickpea plants (*Cicer arietinum* L.) through seed biopriming.	[29]
*B. subtilis* OKB105	Control auxin homeostasis in *A. thaliana.*	[21]
*B. velezensis*, *B. megaterium*,	Produce IAA, gibberellic acid (GA), and siderophore, and solubilize phosphate.	[23]

**Table 2:** Recent research on *Bacillus* spp. as BCAs against different plant pathogens.

*Bacillus* spp. as BCA	Pathogenic Bacterial Species	Treated Plant	References
*Bacillus* sp. USML8 and USML9, and *Bacillus* sp. USMR1,	*Xanthomonas oryzae* pv. *oryzae*	Rice	[48]
*Bacillus* WY66 and WY519	*A. tumefaciens*	Cherry	[36]
*B. amyloliquefaciens* WS-10	*R. solanacearum*	Tobacco	[49]
*B. safensis* ZK-1	*P. syringae* pv. *actinidiae*, *P. alcaligenes* ZK-2, *Clarireedia paspali*	Kiwifruit Turf grass	[50]
*B. subtilis* R31	*R. solanacearum*	Tomato	[51]
*B. subtilis* KA9	*R. solanacearum*	Chili	[52]
*B. subtilis* KJ-2, and *B. amyloliquefaciens* WK-2	*R. solanacearum*	Chili	[53]
*B. velezensis* ZK-3	*X. oryzae* pv. *oryzae*	Rice	[50]
*B. velezensis* JZ	*B. altitudinis m-1*	Strawberry	[35]
*B. velezensis* P64, *B. safensis* P114, and *B. halotolerans* P122	*X. euvesicatoria*	Pepper	[54]
*B. velezensis* Bv21	*X. citri* subsp. *citri*	Onion	[55]
*B. velezensis* JCK-1618, and *B. velezensis* JCK-1696	*Bukholderia contaminans*		[9]
*B*. *velezensis* Y19	*R. solanacearum*	Tobacco	[56]
*B. vallismortis* BL01	*Erwinia carotovora* 3304, *E. carotovora* pv. *atroseptica* 822, *X. campestris* pv. *vesicatoria* 7767, *P. syringae* pv. *tomato* 8949, *P. syringae* pv. *atrofaciens* P-88, *P. syringae* 213	Tomato	[57]
*B. velezensis* FZB42	*Xanthomonas campestris* pv. *campestris*	Cabbage	[58]
	**Pathogenic fungal species**		
*B. amyloliquefaciens* SFB-1	*Ceratocystis fimbriata*	Sweet potato	[38]
*B. amyloliquefaciens* YN201732	*E. cichoracearum*	Tobacco	[42]
*B. atrophaeus* DX-9	*Streptomyces* spp.	Potato	[34]
*B. inaquosorum*, *B. tequilensis*, and *B. spizizenii*	*C. fructicola*	Tea	[59]
*B. paralicheniformis* NB stem 4	*Magnaporthe grisea*	Pearl millet	[60]
*B. siamensis*	*C. gloeosporioides*	Mango	[39]
*B. subtilis*	*Fusarium* spp.	Banana	[61]
*B. subtilis* IBFCBF-4	*F. oxysporum*	Watermelon	[62]
*B. subtilis*	*R. solani, S. rolfsii,* and *F. oxysporum* f. sp. *ciceri*	Chickpea plants	[40]
*B. thuringiensis*	*S. sclerotiorum*	Mustard	[63]
*B. velezensis* Amfr20	*R. solani*, *Verticillium dahliae*, *C. acutatum*, *F. oxysporum* f.sp. *radicis-lycopersici*	Olive	[64]
*B*. *velezensis* Bac302	*Alternaria tenuissima*	Chinese herb (*Schisandra chinensis*)	[65]
*B*. *velezensis* BBE18	*F. oxysporum* f. sp. *cubense*	Banana	[66]
*B. velezensis* Y6	*R. solani*	Rice	[67]
*B. velezensis* LSR7	*Ganoderma pseudoferreum*	Rubber	[68]
*B. velezensis* ZK-3	*Magnaporthe oryzae*	Rice	[50]
*B. velezensis* QSE-21	*B. cinerea*	Tomato	[69]
*B. velezensis* FQ-G3	*B. cinerea*	Tomato	[70]
*B. velezensis* NT35	*Ilyonectria robusta*	Ginseng	[37]
	**Pathogenic parasitic species**		
*B. aryabhattai* Ba1-7, *B. megatherium* Ba2-4, and *B. halotolerans* Ba2-6	SCN (*H. glycines*)	Soybean	[46]
*B. cereus* G5	RKN (*M. graminicola*)	Rice	[47]
*B. licheniformis MW301654*	*M. incognita*	Banana	[71]
*B. megaterium*	RKN (*M. javanica*)	Tomato	[72]
*B. methylotrophicus TA-1*	*M. incognita*	Tomato	[73]
*B. pumilus* S1-10	*M. incognita*	Ginger	[74]
*B. pumilus* Y-26	Stem nematode (*Ditylenchus destructor*)	Sweet potato	[75]
*B. subtilis* JCK-1398	PWN (*Bursaphelenchus xylophilus*)	Pine	[76]
*B. subtilis* JCK-1398	(PWN, *B. xylophilus*)	Pine	[77]
*B. velezensis* Bv-25	*M. incognita*		[78]
*B. velezensis* VB7	RKN (*Meloidogyne incognita*)	Tomato	[44]
*B. velezensis A-27*	*M. incognita*		[79]
*B. velezensis* Ag109	*M. javanica* and *Pratylenchus brachyurus*	Soybean	[80]
	**Pathogenic virial species**		
*B. amyloliquefaciens*	GBNV	Chili	[81]
*B. amyloliquefaciens*	Tomato yellow leaf curl virus (*Begomovirus*)	Tomato	[82]
*B. amyloliquefaciens* TBorg1	TMV (*Tobamovirus*)	Tomato	[83]
*B. amyloliquefaciens*	Tomato-spotted wilt virus (*Tospo virus*)	Tomato	[84]
*B. subtilis* DR06	TMV (*Tobamo virus*)	Tomato	[85]
*B. subtilis* BST8 _+_ *B. subtilis* EBPBS-4 + *B. subtilis* Bbv57	*Orthotospovirus arachinecrosis*	Tomato	[86]
*B. subtilis* BST8, and Bbv57, and *B. amyloliquefaciens* Ka1	GBNV	Tomato	[87]
*B. velezensis* VB7 and *B. licheniformis* Soya1	GBNV	Tomato	[88]
*B. licheniformis*, *B. tequilensis* NBL6, *B. velezensis* VB7	GBNV *Orthotospovirus arachinecrosis*	Cowpea and tomato	[89]

**Table 3:** Commercial BCAs using *Bacillus* spp. available on the market (adopted and modified from [94,95]).

Brand Name	Bacteria Used	Manufacturer	Mode of Action	Target Use	Formulation
RhizoVital	*B. amyloliquefaciens* FZB24	ABiTEP, Gmbh (Berlin, Germany)	PGP	Soil and seed treatment	Liquid
Double nickel	*B. amyloliquefaciens* D747	Certis Biologicals (Columbia, MD, USA)	Antibiosis, ISR induction	Antifungal	Wettable powder (WP)
Stargus, Amplitude	*B. amyloliquefaciens* F727	Marrone Bio Innovations, Bio Ag Services (Faisalabad, Pakistan)	Antibiosis, ISR induction, PGP	Antifungal	WP
Taegro	*B. amyloliquefaciens* FZB24	Novozymes, Salem, VA, USA	Antagonistic activity, ISR	Antifungal	WP
LifeGard WG	*B. mycoides* J	Certis SUA (San Diego, CA, USA)	ISR, fungicides	Broad spectrum antifungal	Wettable granular
Sonata	*B. pumilus* QST2808	Bayer (Leverkusen, Germany)	Produce antifungal compounds	Powdery mildew, rusts	Liquid
Serenade ASO	*B. subtilis* QST713	Bayer (Leverkusen, Germany)	Produce lipopeptides, induces SR	Broad spectrum antifungal	Liquid suspension
Companion	*B. subtilis* GB03	Growth Products, Gustafson Inc., Plano, TX, USA	Antagonistic metabolites, PGP	Turf, ornamentals, vegetables	Liquid
Thuricide	*B. thuringiensis*	Bonide, Southern Ag (Boone, NC, USA)	Toxin production (Cry proteins)	Lepidopteran larvae (insects)	Liquid or dust
Biobit, Dipel	*B. thuringiensis* Subsp. *kurstaki*	Valent BioScienes (Libertyville, IL, USA), Certis (Columbia, MD, USA)	Toxin production (Cry proteins)	Caterpillars (e.g., armyworms, loopers)	WP or DF
XenTari, Agree	*B. thuringiensis* subsp. *Aizawai*	Valent BioSciences (Libertyville, IL, USA), Certis USA	Cry proteins	Diamondback moth, armyworm	WP or DF

**Table 4:** Major VOCs produced by *Bacillus* spp. and their agricultural applications.

VOC Class	Representative Compounds	Major Biological Activity	Target Organism	References
Alcohols	Acetoin, 2,3-butanediol	Plant growth promotion, ISR induction	Enhanced biomass accumulation, modulation of cytokinin, ET and auxin signaling	[14,21]
Alcohols	3-Methyl-2-butanol, 1-Octen-3-ol	Nematicidal activity	Suppression of *M. graminicola* and gall formation	[47]
Ketones	Acetoin (3-hydroxy-2-butanone)	Growth promotion and defense priming	Increased growth and activation of plant defense pathways	[14]
Aldehydes	Formaldehyde-derived metabolic intermediates	Detoxification and ecological fitness	Improved microbial survival in plant-associated environments	[96]
Sulfur-containing compounds	Dimethyl sulfide, dimethyl disulfide	Antifungal and antibacterial activity	Inhibition of fungal and bacterial pathogens	[7,97]
Aromatic/heterocyclic VOCs	Benzothiazole	Antimicrobial activity and defense signaling	Fungal suppression and host defense activation	[7,97]
Mixed VOC blend	Strain-specific VOC mixtures	Root growth promotion and stress adaptation	Altered auxin homeostasis and enhanced root architecture	[18,21]

## Data Availability

No new data were created or analyzed in this study.
